# Supplementary material for: An Effective Sanitizer for Fresh Produce Production: In Situ Plasma-Activated Water Treatment Inactivates Pathogenic Bacteria and Maintains the Quality of Cucurbit Fruit
Source: Microbiol Spectr. 2023 Jul 10;11(4):e00034-23. doi: 10.1128/spectrum.00034-23 (PMC10434273; doi:10.1128/spectrum.00034-23)
Supplement: Supplemental file 1 — Tables S1 to S3 and Fig. S1 to S3. Download spectrum.00034-23-s0001.docx, DOCX file, 1.6 MB [file spectrum.00034-23-s0001.docx]

**Supporting information**

**Table S1.** Estimated characteristic capacitance of the given plasma system and calculated reduced electric field (*E/N*) following Wagner *et al.* (57) where 1 [Td] = 1x10^-17^ Vcm^2^

|  | U_min_ [kV] | C_o_[pF] | C_p_[pF] | U_disch_[kV] | Gap[cm] | E[V/cm] | *E/N*[Td] |
| --- | --- | --- | --- | --- | --- | --- | --- |
| max. current period | 1.25 | 23.5 | 37.3 | 0.767 | 0.1 | 7668.6 | 31.3 |
| max. voltage period | 1.09 | 22.6 | 36.0 | 0.700 | 0.1 | 6696.2 | 27.4 |

**Table S2.** Summarized important gas phase reactions in N_2_/O_2_ plasma system

|  | Process | Rate coefficient [cm^3^s^-1^] [cm^6^s^-1^] ^ǂ^ | Ref. |
| --- | --- | --- | --- |
| Reactions related to atomic oxygen | | | |
| R1 | O + NO + N_2_ → NO_2_ + N_2_ | 1.2×10^-31^(300/T_gas_)^1.8^ | ([1](#_ENREF_1)) |
| R2 | O + NO + NO → NO_2_ + NO | 0.78×1.2×10^-31^(300/T_gas_)^1.8^ | ([1](#_ENREF_1)) |
| R3 | O + NO + O_2_ → NO_2_ + O_2_ | 0.78×1.2×10^-31^(300/T_gas_)^1.8^ | ([1](#_ENREF_1)) |
| R4 | O + NO_2_ + N_2_ → NO_3_ + N_2_ | 8.9×10^-32^(300/T_gas_)^2^ | ([1](#_ENREF_1)) |
| R5 | O + NO_2_ + NO → NO_3_ + NO | 2.4×8.9×10^-32^(300/T_gas_)^2^ | ([1](#_ENREF_1)) |
| R6 | O + NO_2_ + O_2_ → NO_3_ + O_2_ | 8.9×10^-32^(300/T_gas_)^2^ | ([1](#_ENREF_1)) |
| R7 | O + NO → NO_2_ + γ | 4.2×10^-18^, γ indicates photon emission | ([1](#_ENREF_1)) |
| R8 | O + NO_2_ → NO + O_2_ | 9.1×10^-12^(T_gas_/300)^0.18^ | ([1](#_ENREF_1)) |
| R9 | O + NO_3_ → O_2_ + NO_2_ | 1×10^-11^ | ([1](#_ENREF_1)) |
| R10 | O + M → NO + N | Equation S1^§^ for M = N_2_(X), N_2_ (ν_i_) | ([1](#_ENREF_1)) |
| R11 | O + N_2_(A3) → NO + N(2D) | 7.8×10^-12^ | ([2](#_ENREF_2)) |
| R12 | O + N_2_ + M → N_2_O + M | 3.9×10^-35^exp(-10400/T_gas_) | ([1](#_ENREF_1)) |
| R12 | O + N_2_O → NO + NO | 1.5×10^-10^exp(-14090/T_gas_) | ([1](#_ENREF_1)) |
| R13 | O + N + M → NO + M | 1.8×10^-31^(300/T_gas_), M = N, O, NO  1×10^-32^(300/T_gas_)^0.5^ for the rest neutrals | ([1](#_ENREF_1)) |
| R14 | O + O + N_2_ → O_2_ + N_2_ | 2.8×10^-34^exp(720/T_gas_) | ([1](#_ENREF_1)) |
| R15 | O + O + O_2_ → O_2_ + O_2_ | 4.0×10^-33^(300/T_gas_)^0.41^ | ([1](#_ENREF_1)) |
| R16 | O + O + O → O_2_ + O | 3.6×4.0×10^-33^(300/T_gas_)^0.41^ | ([1](#_ENREF_1)) |
| R17 | O + O + N → O_2_ + N | 0.8×4.0×10^-33^(300/T_gas_)^0.41^ | ([1](#_ENREF_1)) |
| R18 | O + O + NO → O_2_ + NO | 0.17×4.0×10^-33^(300/T_gas_)^0.41^ | ([1](#_ENREF_1)) |
| R19 | O + O_2_ + M → O_3_ + M | 7.6×10^-34^(300/T_gas_)^1.9^, M = O_2_, NO, NO_2_, NO_3_ | ([1](#_ENREF_1)) |
|  |  | 3.9×10^-33^*(300/T_gas_)^1.9^, M = N, O | ([1](#_ENREF_1)) |
|  |  | 5.8×10^-34^*(300/T_gas_)^2.8^ for the rest neutrals |  |
| R20 | O + O_2_^-^ → O_3_ + e | 1.5×10^-10^ | ([1](#_ENREF_1)) |
| R21 | O + O^-^ → O_2_ + e | 5.0×10^-10^ | ([3](#_ENREF_3), [4](#_ENREF_4)) |
| Interactions with atomic nitrogen | | | |
| R22 | N + O^-^ → NO + e | 2.6×10^-10^ | ([1](#_ENREF_1)) |
| R23 | N + O_2_^-^ → NO_2_ + e | 5.0×10^-10^ | ([1](#_ENREF_1)) |
| R24 | N + NO → O + N_2_ | 1.8×10^-11^(T_gas_/300)^0.5^ | ([1](#_ENREF_1)) |
| R25 | N + O_2_ → O + NO | 3.2×10^-12^(T_gas_/300)exp(-3150/T_gas_) | ([1](#_ENREF_1)) |
| R26 | N + O_2_(a1) → O + NO | 2×10^-14^exp(-600/T_gas_) | ([5](#_ENREF_5)) |
| R27 | N + NO_2_ → 2O + N_2_ | 9.1×10^-13^ | ([1](#_ENREF_1)) |
| R28 | N + NO_2_ → O + N_2_O | 3×10^-12^ | ([1](#_ENREF_1)) |
| R29 | N + NO_2_ → N_2_ + O_2_ | 7×10^-13^ | ([1](#_ENREF_1)) |
| R30 | N + NO_2_ → NO + NO | 2.3×10^-12^ | ([1](#_ENREF_1)) |
| R31 | N + O_3_ → NO + O_2_ | 2×10^-16^ | ([1](#_ENREF_1)) |
| Interactions between N_x_O_y_ species and O_3_ | | | |
| R32 | NO_2_ + N_2_ → NO + O + N_2_ | 6.8×10^-6^(300/T_gas_)^2^exp(-36180/T_gas_) | ([1](#_ENREF_1)) |
| R33 | NO_2_ + O_2_ → NO + O + O_2_ | 0.78×6.8×10^-6^(300/T_gas_)^2^exp(-36180/T_gas_) | ([1](#_ENREF_1)) |
| R34 | NO_2_ + NO → NO + O + NO | 7.8×6.8×10^-6^(300/T_gas_)^2^exp(-36180/T_gas_) | ([1](#_ENREF_1)) |
| R35 | NO_2_ + NO_2_ → NO + O + NO_2_ | 5.9×6.8×10^-6^(300/T_gas_)^2^exp(-36180/T_gas_) | ([1](#_ENREF_1)) |
| R36 | NO + NO → N + NO_2_ | 3.3×10^-16^(300/T_gas_)^0.5^exp(-39200/T_gas_) | ([1](#_ENREF_1)) |
| R37 | NO + NO → O + N_2_O | 2.2×10^-12^(300/T_gas_)^0.5^exp(-32100/T_gas_) | ([1](#_ENREF_1)) |
| R38 | NO + O_2_ → O + NO_2_ | 2.8×10^-12^exp(-23400/T_gas_) | ([1](#_ENREF_1)) |
| R39 | NO + NO_3_ → NO_2_ + NO_2_ | 1.7×10^-11^ | ([1](#_ENREF_1)) |
| R40 | NO_2_ + NO_2_ → 2NO + O_2_ | 3.3×10^-12^exp(-13500/T_gas_) | ([1](#_ENREF_1)) |
| R41 | NO_2_ + NO_3_ → NO + NO_2_ + O_2_ | 2.3×10^-13^exp(-1600/T_gas_) | ([1](#_ENREF_1)) |
| R42 | NO_3_ + M → NO_2_ + O + M | k_R41_=3.1×10^-5^(300/T_gas_)^2^exp(-25000/T_gas_)  M = N_2,_ O_2,_ NO  10× k_R41,_ M = N, O | ([1](#_ENREF_1)) |
| R43 | NO_3_ + M → NO + O_2_ + M | k_R42_ = 6.2×10^-5^(300/T_gas_)^2^exp(-25000/T_gas_)  M = N_2,_ O_2,_ NO  12× k_R42,_ M = N, O | ([1](#_ENREF_1)) |
| R44 | NO_3_ + NO_2_ → NO + O_2_ + NO_2_ | 8.21×10^-14^exp(-1480/T_gas_) | ([1](#_ENREF_1)) |
| R45 | O_2_ + NO_2_ → NO + O_3_ | 2.8×10^-12^exp(-25400/T_gas_) | ([1](#_ENREF_1)) |
| R46 | NO_2_ + O_3_ → O_2_ + NO_3_ | 1.2×10^-13^exp(-2450/T_gas_) | ([1](#_ENREF_1)) |
| R47 | NO_3_ + NO_3_ → O_2_ + 2NO_2_ | 4.3×10^-12^exp(-3850/T_gas_) | ([1](#_ENREF_1)) |
| R48 | O_3_ + M → O_2_ + O + M | 6.3exp(170/T_gas_) ×6.6×10^-10^exp(-11600/T_gas_),  M = N, O | ([1](#_ENREF_1)) |
|  |  | 0.38×6.6×10^-10^exp(-11600/T_gas_), M = O_2_ | ([1](#_ENREF_1)) |
|  |  | 6.6×10^-10^exp(-11600/T_gas_) for the rest neutrals | ([1](#_ENREF_1)) |
| R49 | NO_3_ + NO_2_ + M → N_2_O_5_ + M | 3.7×10^-30^(T_gas_/298)^-4.1^ | ([1](#_ENREF_1)) |
| R50 | N_2_O_5_ + M → NO_3_ + NO_2_ + M | 1.33×10^-3^(T_gas_/298)^-4.1^exp(-11000/T_gas_) | ([1](#_ENREF_1)) |
| R51 | NO_2_ + NO_2_ + M → N_2_O_4_ + M | 1.44×10^-33^(T_gas_/298)^-3.8^ | ([1](#_ENREF_1)) |
| R52 | N_2_O_4_ + M → NO_2_ + NO_2_ + M | 1.33×10^-5^(T_gas_/298)^-3.8^exp(-6400/T_gas_) | ([1](#_ENREF_1)) |
| R53 | NO + NO_2_ + M → N_2_O_3_ + M | 3.26×10^-34^(T_gas_/298)^-7.7^ | ([1](#_ENREF_1)) |
| R54 | N_2_O_3_ + M → NO + NO_2_ + M | 2.01×10^-7^(T_gas_/298)^-8.7^exp(-4880/T_gas_) | ([1](#_ENREF_1)) |

ǂ All 3-body reactions are in [cm^6^ s^-1^]

^§^ Equation (S1)

M indicates any neutral if not otherwise specified

The gas temperature (T_gas_) and electron temperature are in Kelvin [K] units.

**Table S3.** The assigned coefficient values of a_i_ for the equation S1 in Kelvin ([1](#_ENREF_1)).

$E_{\nu}=3395\nu\left[ 1-6.217\times{10}^{-2}(\nu+1) \right]$

$$k_{\nu}\left( T \right)\left[ {cm}^{3}s^{-1} \right]={\frac{{(E_{\nu}+3000)}^{a_{1}}}{T^{a_{2}}}exp\left( a_{3}+ \frac{38370}{T}a_{4}+\frac{E_{\nu}}{T}a_{5} \right)}$$

| ν | a_1_ | a_2_ | a_3_ | a_4_ | a_5_ |
| --- | --- | --- | --- | --- | --- |
| 0 ≤v≤ 8 | -0.419312 | -0.37836 | -23.04468 | -0.992436 | 0.989385 |
| 9 ≤v≤ 12 | -3.42306 | -1.4234 | 1.423118 | -0.919692 | 0.917323 |
| 13 ≤v≤ 23 | 6.4805404 | -0.279371 | -96.75885 | -0.037869 | 0.019647 |


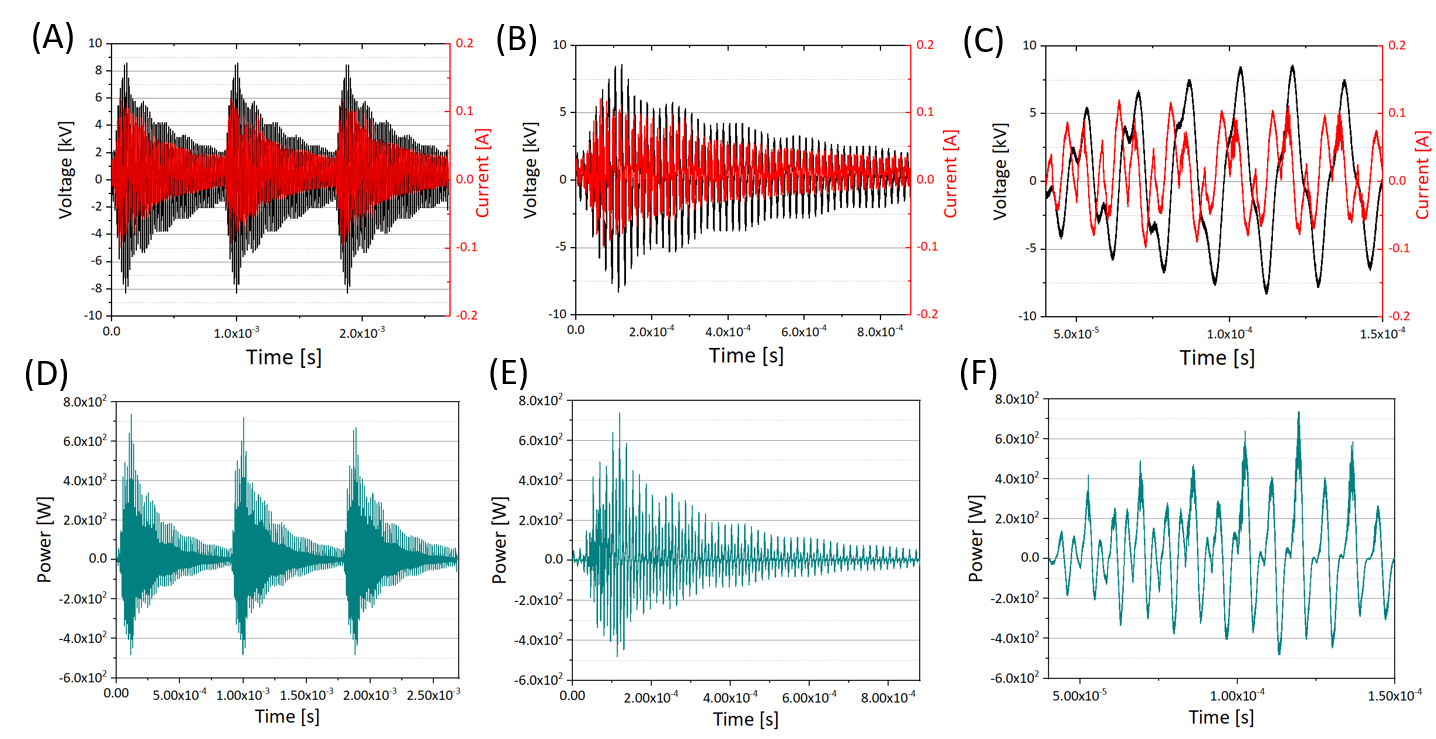


**Figure S1.** The electric waveforms of the measured voltage and current characteristics of the DBDD plasma (A)-(C) and the calculated output power of the DBDD plasma over different time scales (D)-(F).


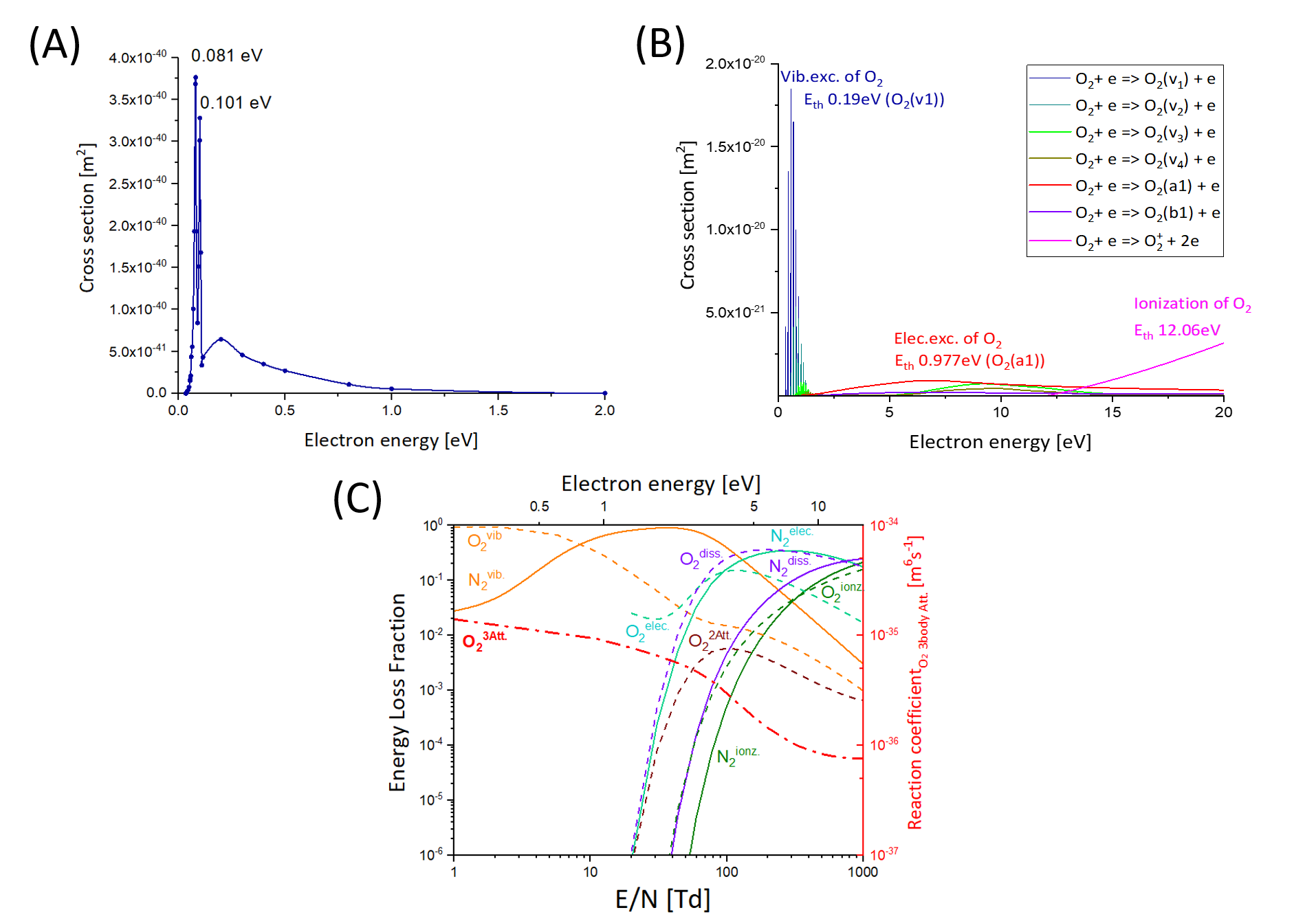


**Figure S2.** The reaction probability and energy loss fractions of electron interactions in air plasma. The cross section data of (A) the 3 body electron attachment to produce O_2_^-^, and (B) other excitation and ionization reactions of oxygen as a function of electron energy, where Vib.exc. and Elec.exc. indicate vibrational excitation and electronic excitation, respectively. The energy loss fraction for different electron interactions in air plasma (C), where the gas composition of N_2_:O_2_=0.8:0.2, 1atm at 300 K. Due to the different physical dimensions of energy loss coefficient of 3 body process [eVm^6^s^-1^] in comparison to 2 body process[eVm^3^s^-1^], this is presented in reaction coefficient [m^6^s^-1^] to show the reduced electric field (*E/N*) and electron energy dependence.


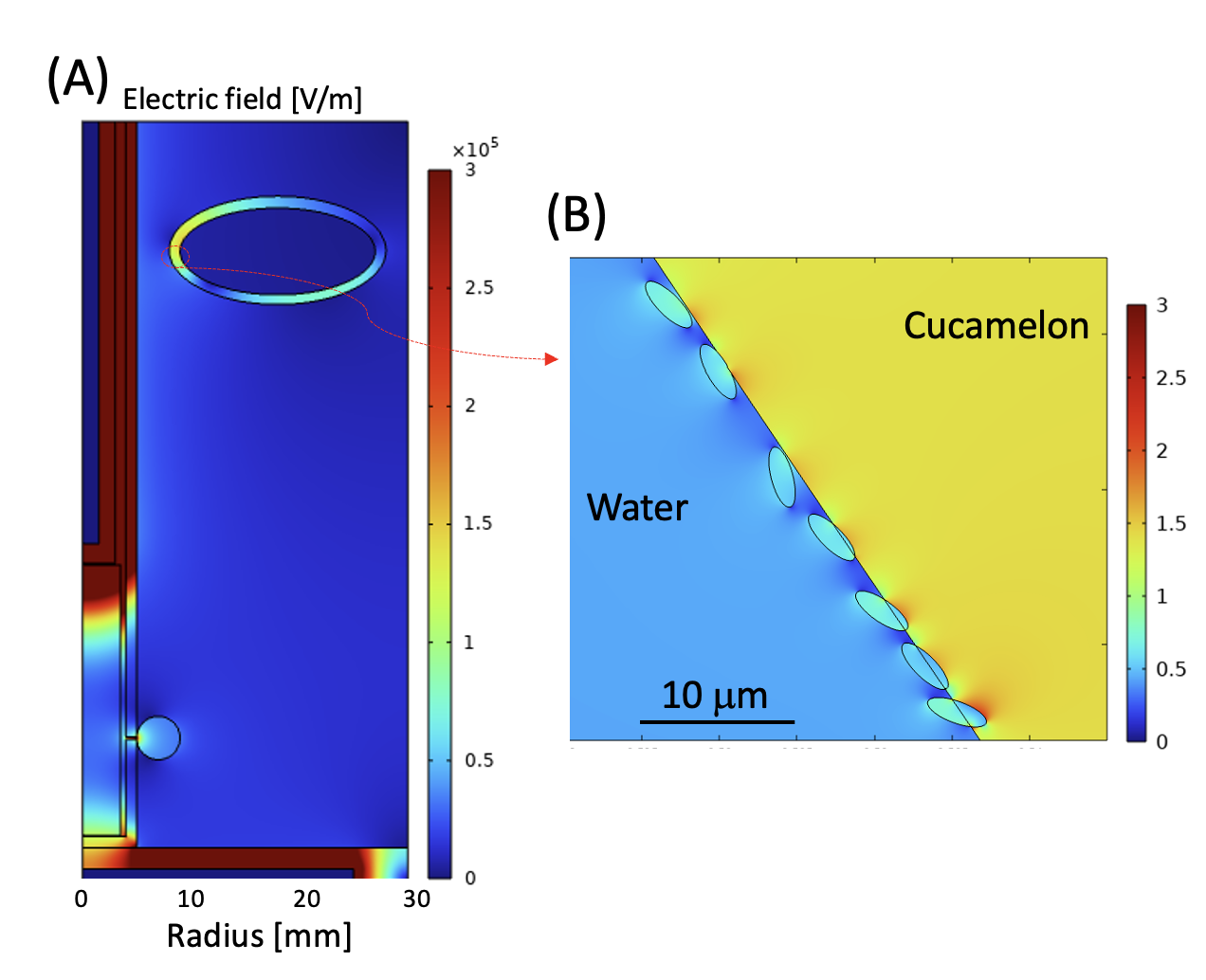


**Figure S3.** Modelling of the electric field distribution for bacterial cells when the cucamelon is positioned towards the top of the water in the DBDD-PAW system. (A) Modelling of the spatial distribution of the normal component of the electric field in the DBDD-PAW system at the peak voltage of 8 kV. (B) The local electric field distribution surrounding the bacterial cells when adhered to the cucamelon surface.

1. M. Capitelli CF, B. Gordiets and A. Osipov. 2000. Plasma Kinetics in Atmospheric Gases. Springer-Verlag Berlin.

2. Kossyi I, Kostinskiy A, Matveyev A, Silakov V. 1992. Kinetic scheme of the non-equilibrium discharge in nitrogen-oxygen mixtures. Plasma Sources Sci Technol 1:207-220. [https://doi.org/10.1088/0963-0252/1/3/011](https://protect-au.mimecast.com/s/VAz5CVARKgC2j73lGikQXw0?domain=doi.org).

3. Itikawa Y, Mason N. 2005. Cross Sections for Electron Collisions with Water Molecules J Phys Chem Ref Data 34:1-22. [https://doi.org/10.1063/1.1799251](https://protect-au.mimecast.com/s/LPC9CWLVXkU6w2OjJt1SwT_?domain=doi.org).

4. Stafford DS, Kushner MJ. 2004. O2(Δ1) production in He∕O2 mixtures in flowing low pressure plasmas. Journal of Applied Physics 96:2451-2465. [https://doi.org/10.1063/1.1768615](https://protect-au.mimecast.com/s/7JNFCXLW2mU4712nwtM2_CG?domain=doi.org).

5. Gordiets BF, Ferreira CM, Guerra VL, Loureiro JMAH, Nahorny J, Pagnon D, Touzeau M, Vialle M. 1995. Kinetic model of a low-pressure N/sub 2/-O/sub 2/ flowing glow discharge. IEEE Transactions on Plasma Science 23:750-768. [https://doi.org/10.1109/27.467998](https://protect-au.mimecast.com/s/eFwaCYW8NocDoQM3rf2HFqS?domain=doi.org).
